# Supplementary material for: Individual patterns of functional connectivity in neonates as revealed by surface-based Bayesian modeling
Source: Imaging Neurosci (Camb). 2025 Mar 20;3:imag_a_00504. doi: 10.1162/imag_a_00504 (PMC12319736; doi:10.1162/imag_a_00504)
Supplement: Supplementary Material [file imag_a_00504-supp.pdf]

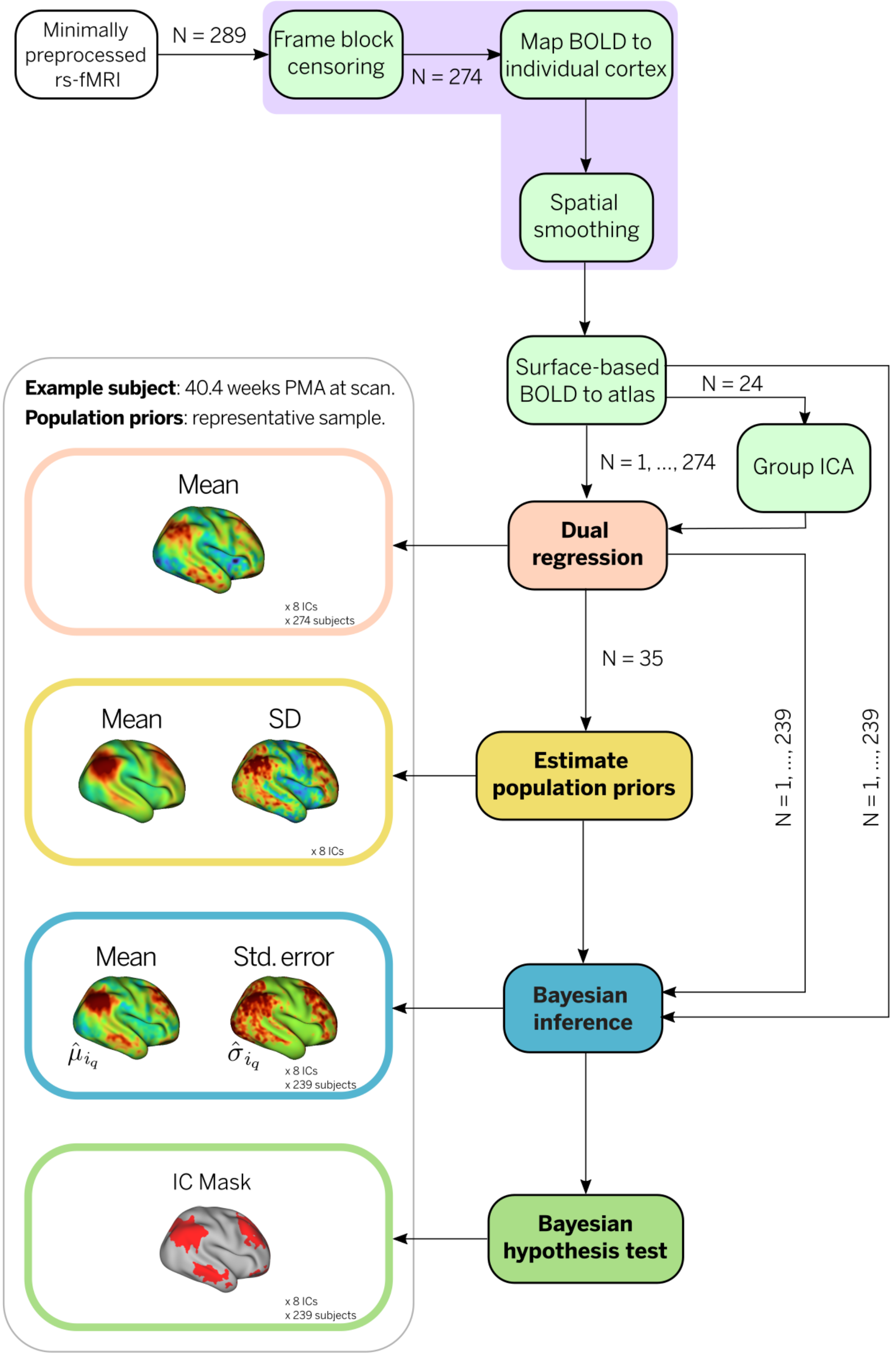

Figure S1: Outline of the processing steps. The minimally preprocessed BOLD volumes were projected onto their corresponding cortical surface and subsequently mapped to atlas space using spherical alignment. Group ICA maps were estimated from a subset of 24 neonates (age at scan: 43.5 - 45 weeks PMA). Rough estimates of individual IC maps were obtained for the whole cohort using dual regression. A population template (mean and inter-subject variance) was obtained from 35 subjects, uniformly sampled based on age at scan to provide an unbiased representation of the population. Using Bayesian inference, individual maps of mean and variance were estimated for all infants, excluding the 35 subjects considered in the estimation of the population priors. Individual t-statistic maps were estimated for each subject and significant areas of engagement were identified via Bayesian hypothesis test ($p < 0.01$).

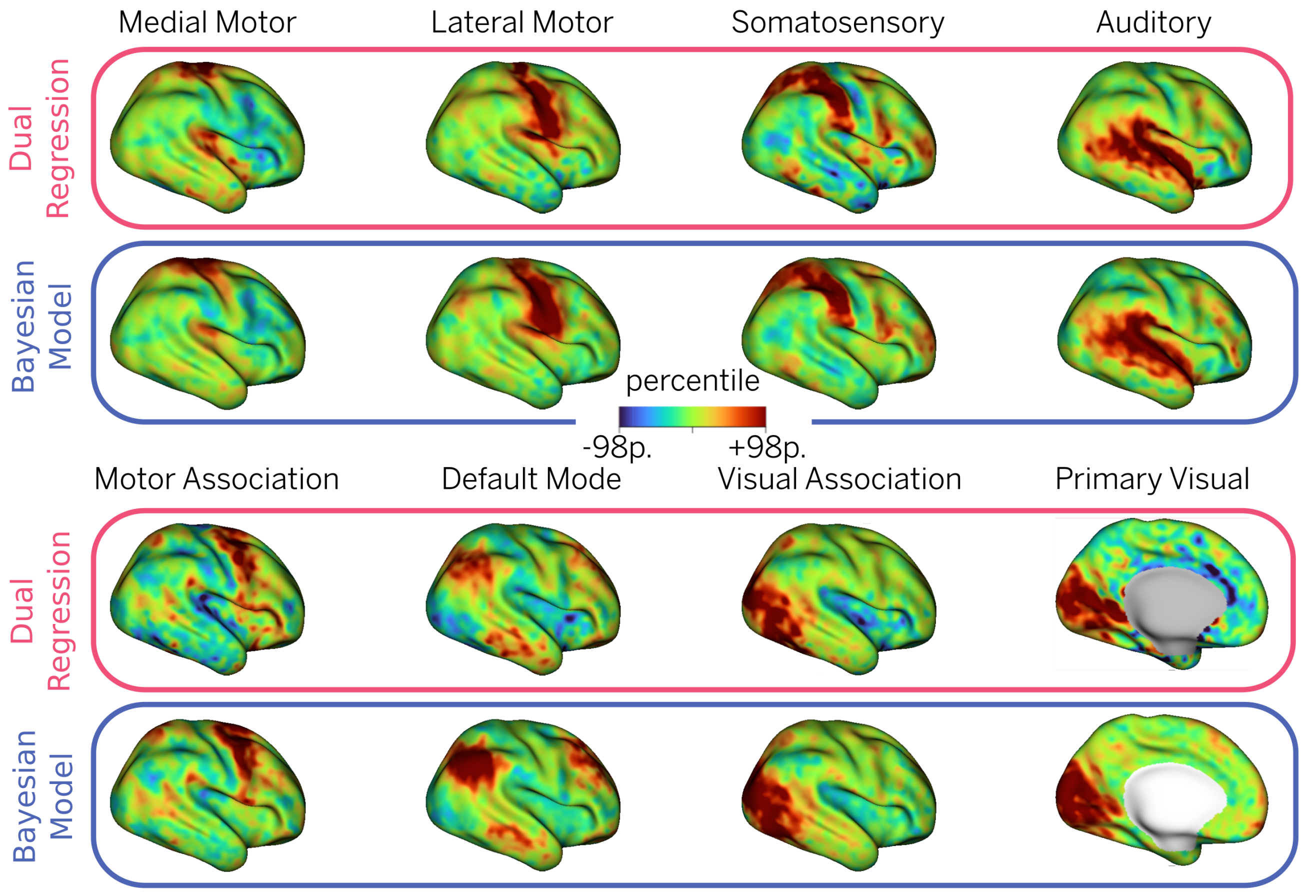

Figure S2: Full view of cortical t-statistic maps for a single subject. Eight networks obtained for a term-born neonate (age at birth: 40 weeks PMA) scanned at 42.6 weeks PMA. All networks were projected onto an inflated 40-week surface atlas.

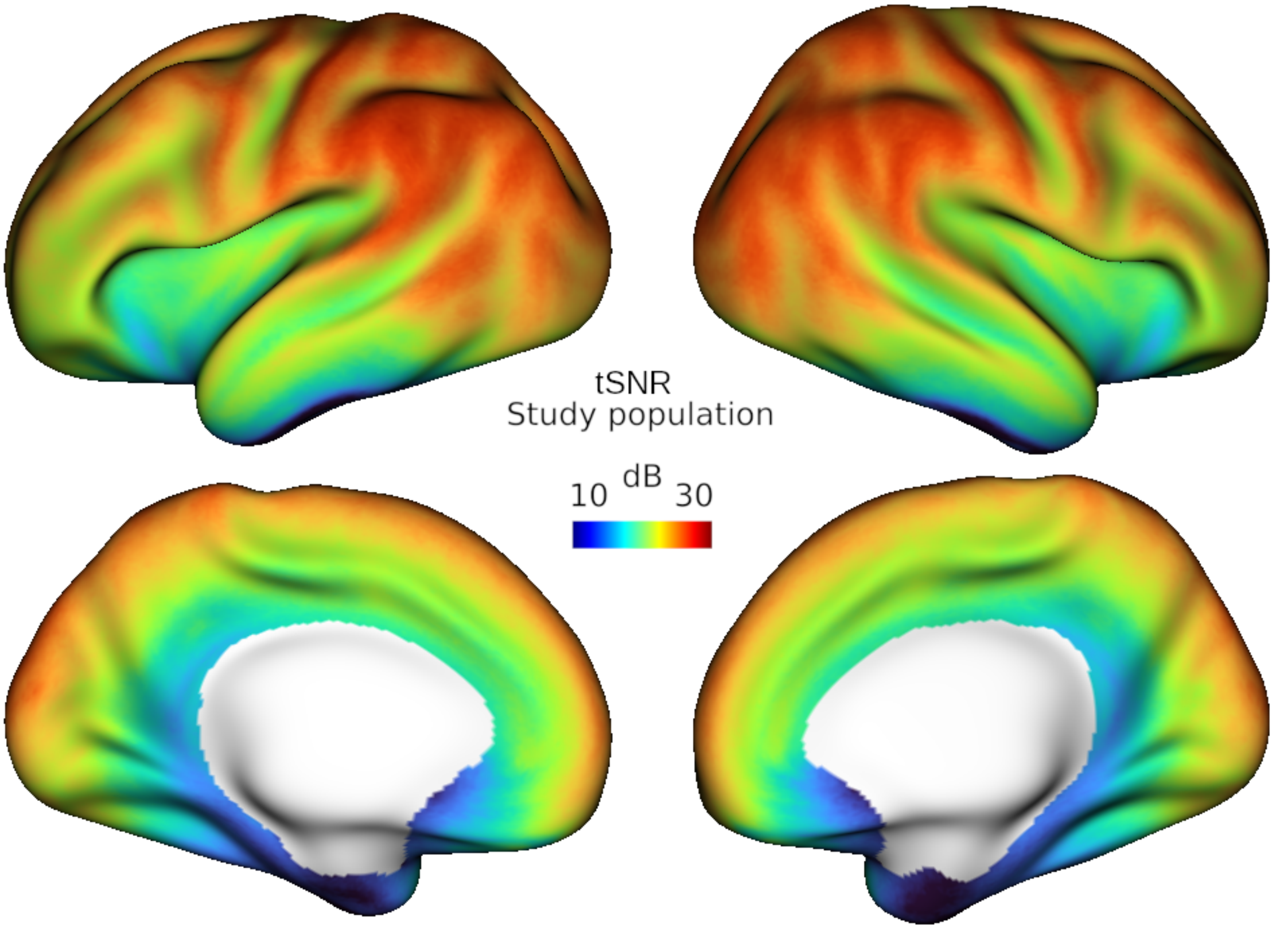

Figure S3: Global tSNR. Temporal signal-to-noise ratio (tSNR) for the entire cohort (N = 289) computed as the ratio between the mean BOLD signal and the mean standard deviation at each vertex. tSNR was calculated after data preprocessing (including frame censoring, volume-to-surface mapping and spatial smoothing) and projected onto the inflated 40-week surface atlas.

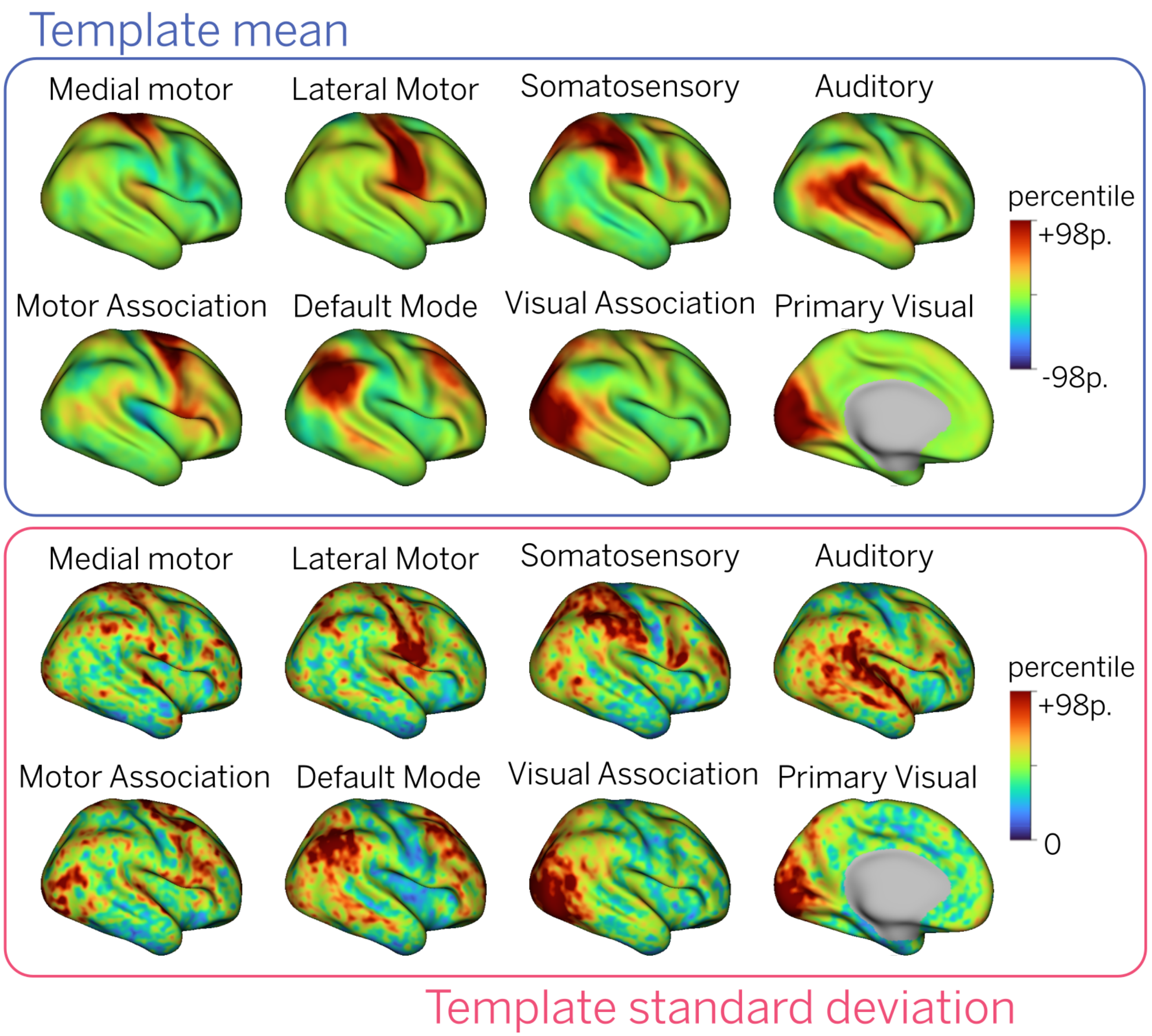

Figure S4: Empirical population priors or template for eight RSNs. Mean and standard deviation maps obtained from a representative subset of term-born infants from the dHCP database. All maps were projected onto an inflated 40-week surface atlas.

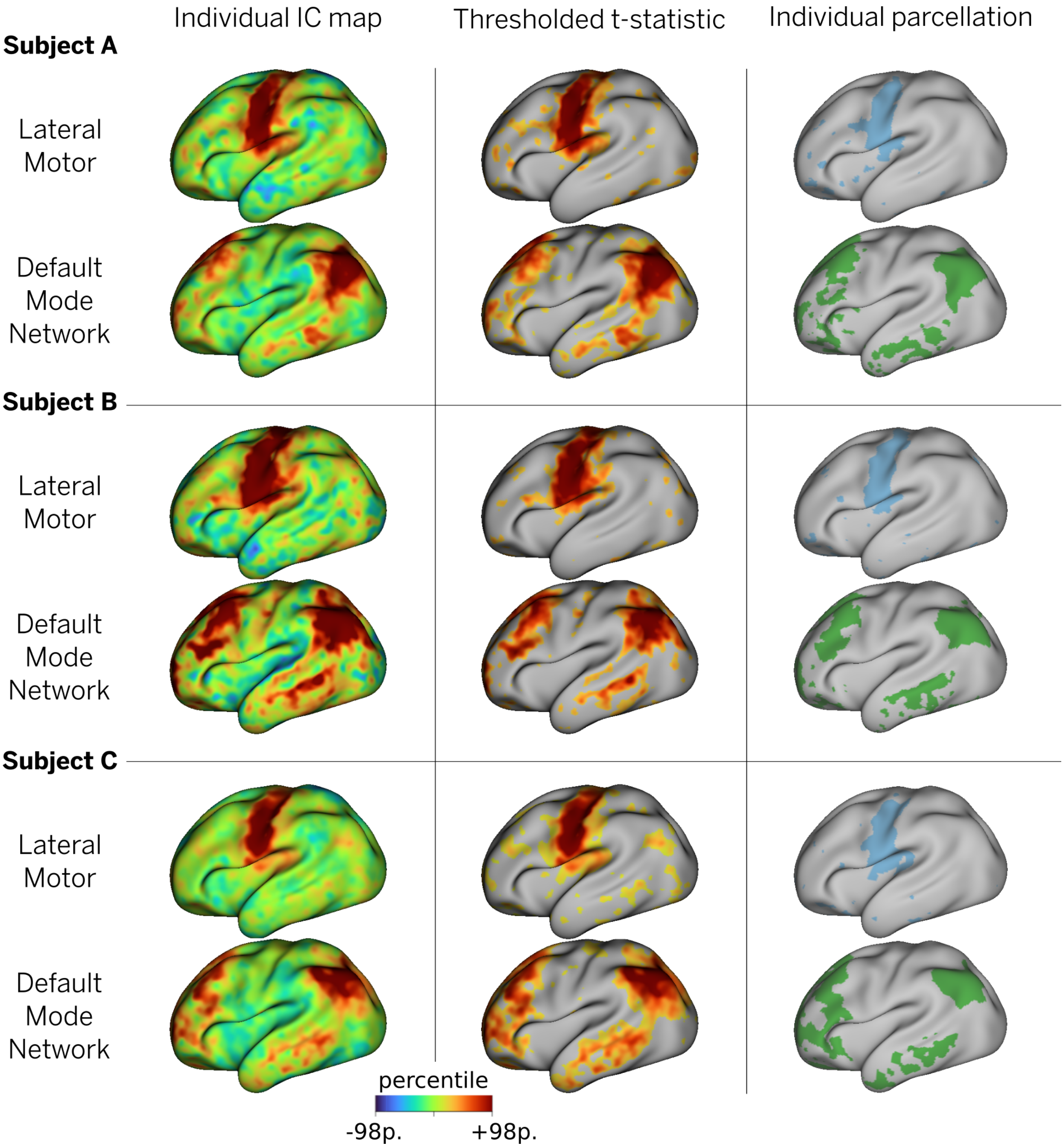

Figure S5: Intermediate steps of the Bayesian analysis in three different subjects. The unthresholded individual mean IC map (left) shows their corresponding probability maps (represented as a thresholded t-statistic map) after a Bayesian hypothesis test using significance level $\alpha = 0.01$ (center) and their corresponding individual winner-takes-all parcellation (right).

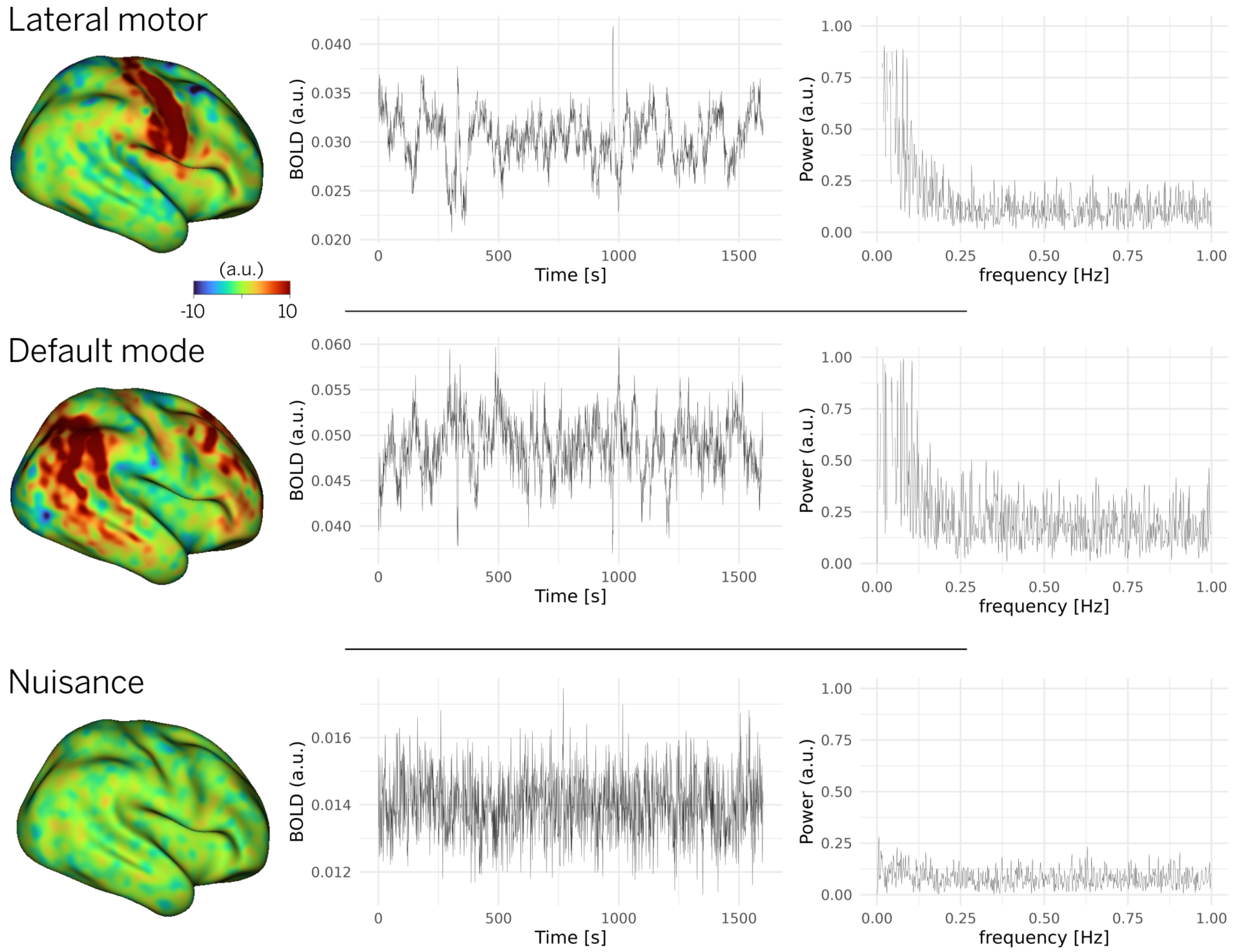

Figure S6: Time courses and spectra of signal and noise components. Spatial maps of three different ICs associated with networks and nuisance (left), their associated time course (center), and power spectrum (right).

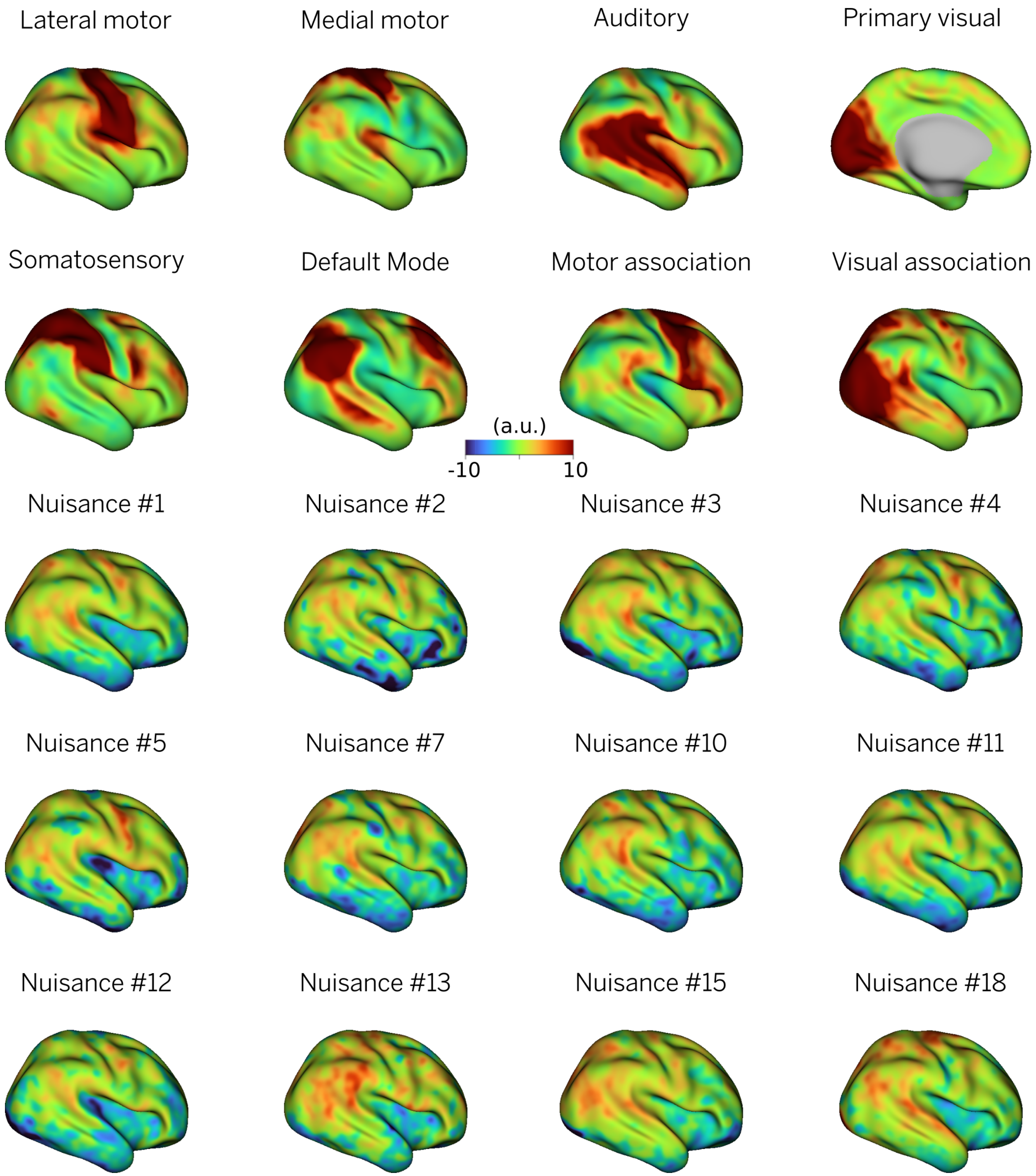

Figure S7: Maps of signal and nuisance components. Twenty group-level IC maps were obtained from a sample of 24 subjects (age at scan 43.5 - 44.5 PMA). All maps are projected onto the inflated 40-week atlas for visualization purposes.

Resting-state network	r	Uncorrected p-value	Corrected p-value
Medial motor	0.300	**1.1E-06**	**9.1E-06**
Lateral motor	0.205	**9.9E-04**	**7.9E-03**
Somatosensory	0.234	**1.6E-04**	**1.3E-03**
Auditory	0.250	**5.8E-05**	**4.6E-04**
Primary visual	0.133	**3.4E-02**	2.7E-01
Default mode	0.289	**2.9E-06**	**2.3E-05**
Motor association	0.274	**9.2E-06**	**7.4E-05**
Visual association	0.244	**8.4E-05**	**6.7E-04**

Table S1: Effect of age at scan. Spearman's r (1st column) and p-values before (2nd column) and after (3rd column) Bonferroni correction for multiple comparisons across the different resting-state networks. Results in bold indicate a statistically significant relationship between age and connectivity strength ($p < 0.05$).
